# Notes and Notices

**Published:** 1883-08

**Authors:** 


### NOTES AND NOTICES.

QUERY No. 1.—If Sol., Luna, etc., can be potentized for use on this mundane sphere, why cannot "Hell" be potentized for use *hereafter?* A high potency antidotes a *low* one!

QUERY No. 2.—Why is a rooster on a neighbor's fence like a young Doctor's office? No business there!

THE I. H. A.—An interesting meeting of the International Hahnemannian Association was held at Niagara. Hereafter, the Association will meet three days in advance of the Institute, but at same place.

Officers for 1884: President, George F. Foote, M. D.; Vice-president, Dr. J. P. Mills; Secretary, Dr. Custis; Treasurer, Dr. Cranch.

BOOKS AS CONVEYERS OF CONTAGION.—"There is no more powerful apparatus for the conveyance of disease than a book," says the London *Lancet.* A list of the maladies most easily conveyed by means of books is given as follows: "Measles, scarlet fever, diphtheria, sore throat, whooping cough, bronchitis, and perhaps phthisis." The germs of disease "may lie for weeks, months, or perhaps years, between the pages of a bound book, to be dislodged at some unpropitious moment when the volume chances to be handled by a susceptible person."

DR. ADOLPH FELLGER sailed July 11th, via the steamship Elbe, for *Bremen,* for a trip of three months. As Dr. Fellger has labored continuously (and most successfully) in this city for thirty-six years, he both needs and deserves a rest; and none could be more appropriate than a visit to the "Fatherland." Prior to his departure, Dr. Fellger was entertained by some of his professional associates. Gentleman from New York and Boston were invited to join in this tribute of respect and affection to our honored colleague.

DROWNING OF TWO PHYSICIANS.—A most distressing accident occurred at Reading, Pa., July 6th. Two prominent homœopathic physicians, Drs. A. C. and W. A. Detweiler, of that city, were accidentally drowned while bathing in the Schuylkill River near that place. These gentlemen were engaged in a fine practice, and were much respected by all. Both were graduates of Jefferson Medical College of Philadelphia.

DR. LOUIS DE HYSERN, of Madrid, died recently. He was, with the late Dr. Nunez, very active in introducing Homœopathy into Spain.

